# Enhanced oxidative stress by alcohol use in HIV+ patients: possible involvement of cytochrome P450 2E1 and antioxidant enzymes

**DOI:** 10.1186/s12981-015-0071-x

**Published:** 2015-09-22

**Authors:** Anusha Ande, Namita Sinha, P. S. S. Rao, Carole P. McArthur, Leo Ayuk, Paul N. Achu, Annette Njinda, Anil Kumar, Santosh Kumar

**Affiliations:** School of Pharmacy, University of Missouri-Kansas City, Kansas City, MO 64108 USA; College of Pharmacy, University of Tennessee Health Science Center, 881 Madison Ave, Memphis, TN 38163 USA; School of Dentistry, University of Missouri-Kansas City, Kansas City, MO 64108 USA; Regional Hospital, Box 818, Bamenda, North West Province Cameroon; Mezam Polyclinic HIV/AIDS Treatment Center, Bamenda, Cameroon

**Keywords:** Alcohol, HIV, Oxidative stress, Cytochrome P450, Antioxidant enzymes, Cytokines

## Abstract

**Background:**

Alcohol consumption is prevalent amongst HIV positive population. Importantly, chronic alcohol use is reported to exacerbate HIV pathogenesis. Although alcohol is known to increase oxidative stress, especially in the liver, there is no clinical evidence that alcohol increases oxidative stress in HIV positive patients. The mechanism by which alcohol increases oxidative stress in HIV positive patients is also unknown.

**Methods:**

To examine the effects of alcohol use on oxidative stress we recruited HIV+ patients who reported mild-to-moderate alcohol use. Strict inclusion and exclusion criteria were applied to reduce the effect of other therapeutic drugs metabolized via the hepatic system as well as the effect of co-morbidities such as active tuberculosis on the interaction between alcohol and HIV infection, respectively. Blood samples were collected from HIV-negative alcohol-users and HIV positive alcohol-users followed by collection of plasma and isolation and fractionation of monocytes from peripheral blood. We then determined oxidative DNA damage, glutathione level, alcohol level, transcriptional level of cytochrome P450 2E1 (CYP2E1) and several antioxidant enzymes, and plasma level of cytokines.

**Results:**

Compared to HIV-negative alcohol users, HIV-positive alcohol users demonstrated an increase in oxidative DNA damage in both plasma and CD14+ monocytes, as well as, a relative increase in oxidized/reduced glutathione (GSSG/GSH) in plasma samples. These results suggest an increase in oxidative stress in HIV-positive alcohol users compared with HIV-negative alcohol users. We also examined whether alcohol metabolism, perhaps by CYP2E1, and antioxidant enzymes are involved in alcohol-mediated increased oxidative stress in HIV-positive patients. The results showed a lower *plasma* alcohol level, which was associated with an increased level of CYP2E1 mRNA in *monocytes*, in HIV-positive alcohol users compared with HIV-negative alcohol users. Furthermore, the transcription of major antioxidants enzymes (catalase, SOD1, SOD2, GSTK1), and their transcription factor, Nrf2, were reduced in monocytes obtained from HIV positive alcohol users compared to the HIV-negative alcohol user group. However, no significant change in levels of five major cytokines/chemokines were observed between the two groups.

**Conclusions:**

The data suggests that alcohol increases oxidative stress in HIV+ patients, perhaps through CYP2E1- and antioxidant enzymes-mediated pathways. The enhanced oxidative stress is accompanied by a failure of cellular antioxidant mechanisms to maintain redox homeostasis. Overall, the enhanced oxidative stress in monocytes may exacerbate HIV pathogenesis in HIV positive alcohol users.

## Background

Chronic alcohol consumption is known to increase the incidence of acquiring HIV infection due to elevated propensity for risky sexual practices following drinking [1]. On the other hand, according to the National Institute on Alcohol Abuse and Addiction (NIAAA), people infected with HIV are twice as likely to consume alcohol than the general population [2]. Several studies have reported significantly higher rates of alcohol abuse/alcohol use disorder in HIV-infected patients compared to the general population [3]. The consumption of alcohol amongst HIV patients is known to enhance HIV pathogenesis and disease progression, especially in patients who are not receiving antiretroviral therapy (ART) yet [4–6]. Moreover, studies with non-human primates have shown increased viral load upon alcohol consumption [7].

The exact mechanisms that govern the interaction between alcohol and HIV pathogenesis remains unclear. An in vitro study has suggested a possible role of oxidative stress in increasing HIV replication [8]. However, there is no clinical data on the in vivo involvement of alcohol-mediated oxidative stress in HIV replication. Our previous studies have demonstrated the possible involvement of cytochrome P450 2E1 (CYP2E1)-mediated alcohol-induced oxidative stress in HIV systems including blood monocytes [9, 10]. CYP2E1 is the major CYP isoform responsible for the metabolism of alcohol in chronic users, and is induced following chronic alcohol exposure [11, 12]. The metabolism of alcohol by CYP2E1 is known to generate reactive oxygen species (ROS), which eventually increases oxidative stress in the cells, especially those in the liver [13]. Thus, CYP2E1-mediated oxidative stress has been implicated in modulating the harmful effects of alcohol in the liver [14].

In addition to CYP2E1-mediated increased oxidative stress by alcohol, a decreased level of antioxidant enzymes (AOEs) may also cause an increase in alcohol-mediated oxidative stress [15]. Catalase, cytoplasmic superoxide dismutase 1 (SOD1), and mitochondrial SOD2 are the major AOEs that play an important role in detoxifying ROS and maintaining cellular oxidative homeostasis [16]. Similarly, glutathione S-transferase kappa 1 (GSTK1) is a mitochondrial antioxidant enzyme responsible for protecting mitochondrial components against oxidative stress [17].

In this study, we examined the effects of alcohol use in HIV positive patients through possible involvement of oxidative stress and mediators that produce oxidative stress in HIV positive alcohol users using clinical samples from well-characterized patients. We collected plasma and monocytes from HIV positive alcohol users and studied the levels of oxidative stress markers, alcohol metabolism, CYP2E1, various AOEs, and cytokines/chemokines. In addition to plasma collected, we fractionated CD14+ monocytes for this study because they are the known cellular targets of HIV infection and serve as major viral reservoir, especially in the brain [18].

## Results and discussion

### Study subjects

This study was designed to examine the involvement of oxidative stress, mediated through CYP and AOE pathways, by alcohol use in ART-naïve HIV-infected individuals. As summarized in Table 1, in this study, following the application of rigorous inclusion and exclusion criteria, we recruited 10 subjects (six HIV negative alcohol users and four HIV-positive alcohol users). The average age for the groups were 37 (for HIV-ve ALC) and 40 (for HIV+ve ALC) and the median age was 37 (for HIV-ve alcohol users) and 39 (for HIV+ve alcohol users). The ratio of men to women in HIV-ve ALC group was 1:1 (3 male and 3 female) and in HIV+ve ALC was 1:3 (1 male and 3 female). The average CD4+ count for HIV negative and HIV positive alcohol user groups was 681 and 418, respectively. The average viral load was 10.9 ± 8.1 log copies/ml (Table 1). Our analysis of subject-to-subject variation did not show significant difference in CD4+ counts, alcohol levels, and the levels of CYP, AOEs, and cytokines/chemokines on the basis of age and gender differences (data not shown). This study design and analysis with respect to subject’s demographic data are similar to our recent studies performed in HIV positive smokers [19, 20].

**Table 1:** Demographics and clinical findings for the recruited subjects

Subjects	HIV negative alcoholics	HIV positive alcoholics
Number, age (years), male/female ratio		
Number	6	4
Age range	32–51	37–42
Median age	35	38
Male/female	1	0.33
CD4 count (cells/µL)		
Range	576–836	321–480
Mean ± SE	681 ± 39	418 ± 35
Viral load (log copies/mL)		
Range		0.06–35
Mean ± SE		10.9 ± 8.1

### Enhanced oxidative stress in HIV+ alcohol users

**Fig. 1:**
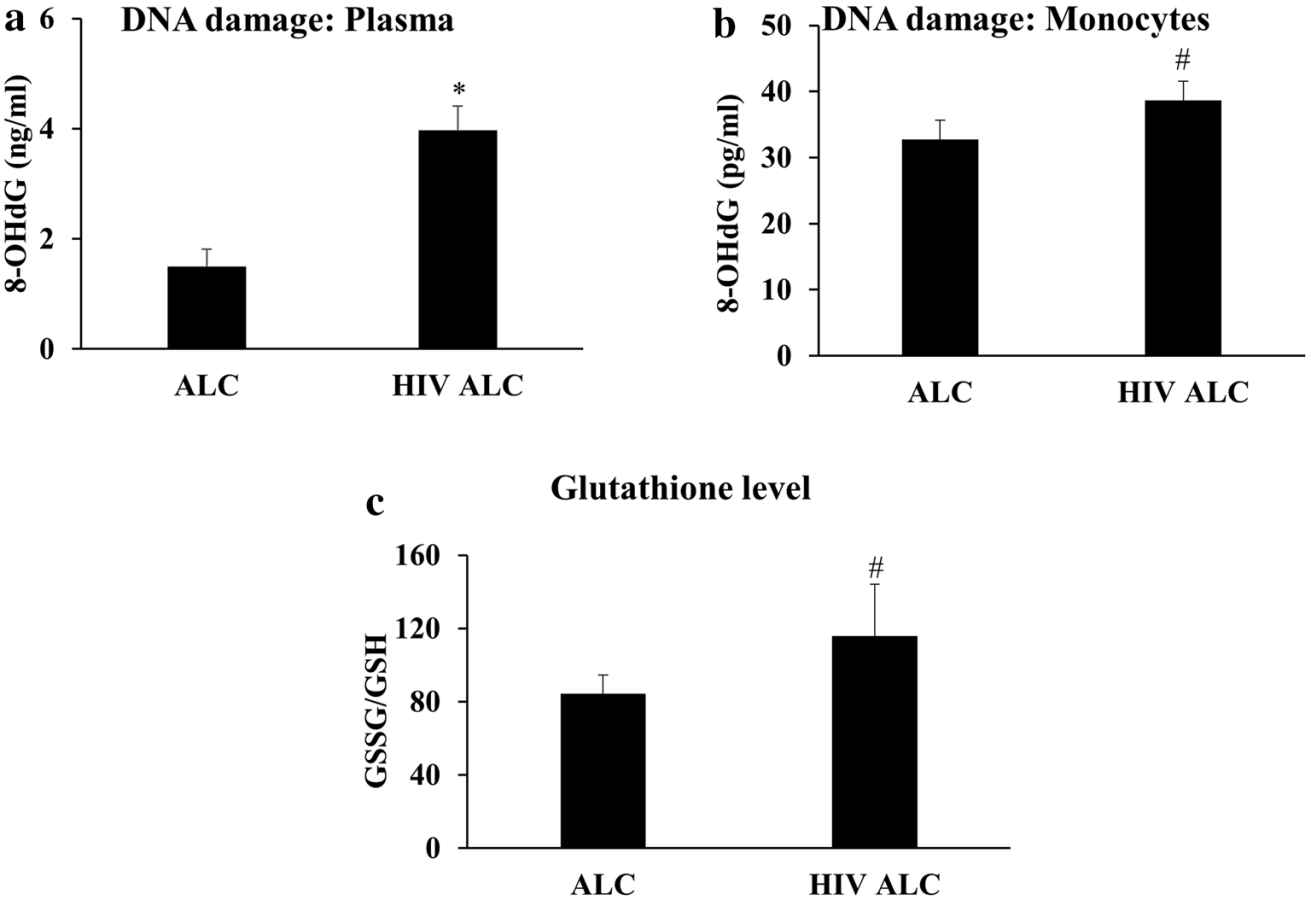
Oxidative stress parameters in HIV negative alcohol users (ALC; n = 6) and HIV positive alcohol users (HIV+ ALC; n = 4). **a** Oxidative DNA damage in plasma. **b** Oxidative DNA damage in monocytes. **c** Ratio of oxidized glutathione to reduced glutathione level (GSSG/GSH) in plasma. p ≤ 0.1 (^#^) and p ≤ 0.05 (*) represent *borderlin*e significance and significance, respectively

Although alcohol is known to increase oxidative stress, especially in the liver [14], there is no clinical evidence reported of increased oxidative stress in the *plasma* and *monocytes* from the HIV positive alcohol users. Results from this study showed significantly enhanced levels (~2.5-fold) of oxidative DNA damage (8-OHdG) in plasma collected from the HIV positive alcohol user group as compared to the HIV negative alcohol user group (Fig. 1a; p ≤ 0.05). Similarly, a trend towards significantly increased oxidative DNA damage was also observed in *monocytes* collected from alcohol users who were infected with HIV in comparison to the cells from uninfected alcohol users (Fig. 1b; p ≤ 0.1). In addition to oxidative DNA damage, the ratio of oxidized and reduced *plasma* glutathione (GSSG/GSH) was elevated (Fig. 1c; p ≤ 0.1) in HIV positive alcohol users compared to the uninfected alcohol user control group.

Oxidative DNA damage is a known marker of oxidative stress [21]. An increase in its level, both in plasma and monocytes, clearly suggests an increased oxidative stress. While monocytic oxidative DNA damage is specific to an increase in oxidative stress in monocytes, an increase in plasma DNA damage may reflect an increase in oxidative stress in other cellular systems such as liver and other blood cells. Compared with the data for HIV-infected subjects, presented in our earlier publication [19], alcohol use clearly exacerbates the plasma DNA damage in HIV-infected alcohol users. This observation indicates a role of alcohol-, and possibly CYP2E1-, mediated changes in oxidative stress in HIV infected alcohol users.

Glutathione, on the other hand, is a known scavenger of ROS and serves as an important antioxidant [22]. Chronic alcohol consumption is known to be associated with reduced plasma glutathione levels compared to healthy subjects [23]. Ratio of levels of oxidized glutathione to reduced glutathione (GSSG/GSH) represents the redox status of the body and an increase in this ratio suggests a possible compensatory antioxidant mechanism to counter the enhanced oxidative stress. The change in GSSG/GSH ratio observed in this study, in addition to enhanced oxidative DNA damage in plasma and monocytes, further supports the existence of increased oxidative stress in the HIV positive alcohol users compared to the HIV-negative alcohol users.

### Altered plasma alcohol level in HIV-infected patients

**Fig. 2:**
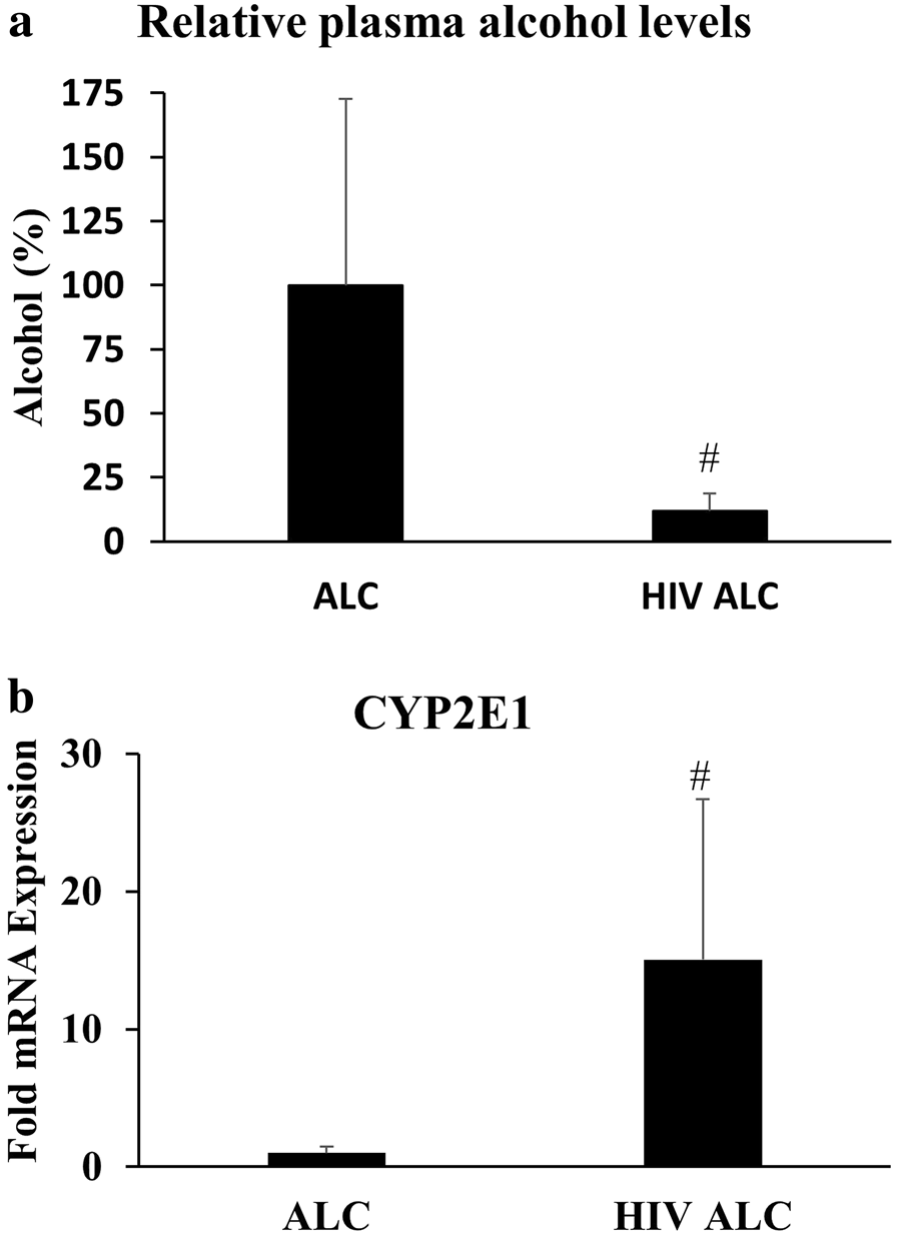
Relative plasma alcohol level (**a**) and CYP2E1 mRNA level in monocytes (**b**) in HIV positive alcohol users (HIV+ ALC; n = 4) in comparison to HIV negative alcohol users (ALC; n = 6). p ≤ 0.1 (^#^) represents *borderline* significance. 100 % alcohol in the plasma of ALC subjects corresponds to 7.1 mM

Although alcohol dehydrogenase is the major alcohol metabolizing enzyme in acute alcohol users, expression/activity of CYP2E1 is known to be induced (up to 10 fold) following extended exposure to alcohol [24, 25]. Thus, CYP2E1 is a major alcohol metabolizing enzyme in chronic alcohol users. Furthermore, CYP2E1-mediated alcohol metabolism is known to increase ROS, especially in the liver, leading to liver toxicity [13, 14]. However, it is not known whether alcohol metabolism is altered in HIV-infected alcohol users and whether CYP2E1 plays any role in it. In this study, plasma alcohol concentration was found to be reduced by ~eightfold (Fig. 2a; p ≤ 0.1) in HIV-infected alcohol users as compared to HIV-uninfected alcohol users, suggesting enhanced alcohol metabolism as a possible mechanism. Importantly, this observation was associated with a significant trend towards enhanced transcription of CYP2E1 (~eightfold) in monocytes collected from HIV-infected alcohol users as compared to HIV-uninfected alcohol users (Fig. 2b; p ≤ 0.1).

Previously, our in vitro studies have demonstrated the presence of CYP2E1 in monocytes and its role in alcohol metabolism in these cells [9, 26]. Although hepatic CYP2E1 is the major player in alcohol metabolism, reflected by plasma alcohol level, *monocytic* CYP2E1 also appears to play a role in alcohol metabolism. Future studies employing a controlled alcohol intake paradigm in a larger group of HIV+ patients using a longitudinal design would help confirm the role of CYP2E1-mediated enhanced metabolism in HIV-infected alcohol users. Moreover, since monocytes are known targets for HIV infection, it is possible that the adverse effects of alcohol on monocytes are due to increased expression of monocytic CYP2E1. While our previous in vitro studies have elucidated the cellular mechanisms for alcohol-mediated changes in monocytes [9, 10, 26], the current finding represents the first ex vivo evidence suggesting possible involvement of CYP2E1 in HIV positive alcohol users. Since CYP2E1 is not present at significant levels in lymphocytes [26], the role of monocytic CYP2E1 may be important in governing the effects of alcohol on HIV pathogenesis.

### Attenuated transcription of major AOEs and regulating transcription factor

**Fig. 3:**
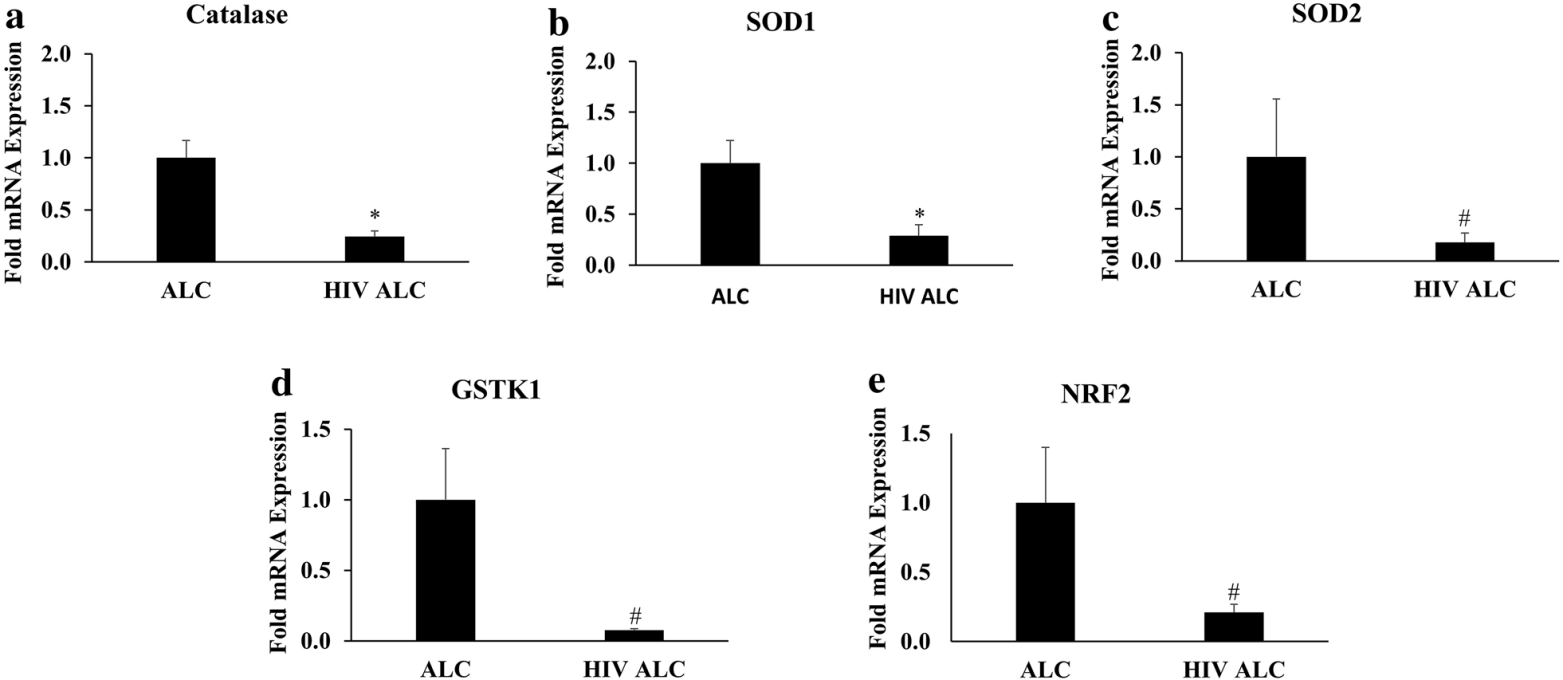
Relative mRNA levels of AOEs and transcription factor. **a** Catalase, **b** SOD1, **c** SOD2, **d** GSTK1, and **e** Nrf2 in HIV positive alcohol users (HIV+ ALC; n = 4) in comparison to HIV negative alcohol users (ALC; n = 6). p ≤ 0.1 (^#^) and p ≤ 0.05 (*) represent *borderline* significance and significance, respectively

In addition to CYP2E1-mediated increased alcohol metabolism, the expression of AOEs play a critical role in governing oxidative stress [16]. In this study, alcohol users with existing HIV infection showed significantly reduced (~fourfold) transcription of catalase (Fig. 3a) and SOD1 (Fig. 3b) compared to uninfected alcohol users (p ≤ 0.05). Moreover, compared to the cells from uninfected alcohol users, monocytes obtained from the HIV-infected alcohol users displayed a trend towards reduced (p ≤ 0.1) transcription of other AOEs; SOD2 (Fig. 3c) and GSTK1 (Fig. 3d). Importantly, these observations were associated with a trend towards significant reduction in mRNA levels (p ≤ 0.1) for the major transcription factor responsible for AOE expression in cells such as Nrf2 (Fig. 3e).

Alcohol consumption is known to attenuate antioxidant capacity by decreasing the expression of AOEs and its transcription factor NRF2. Levels of SOD enzymes, for instance, were found to be reduced following alcohol administration in animal models [27]. Similarly, HIV infection has been shown to impact the activity/expression of AOEs [28]. The direct impact of HIV infection is the enhanced production of superoxide anion in phagocytic cells, presumably due to altered expression/activity of SOD enzymes [29, 30]. The results from the present study suggest a further compromised expression of AOEs in patients exposed to both HIV and alcohol. Additionally, owing to decreased expression of GSTK1 in the HIV+ve ALC group, our data from this study suggests an increased probability for alcohol-mediated mitochondrial injury in HIV-infected alcohol users as compared to alcohol users not infected by HIV. Moreover, a comparison between AOEs and NRF2 expression between HIV-ve infected subjects [19] and the HIV+ve ALC group (Fig. 3) suggests an overall decrease in transcription of antioxidant genes in HIV-infected alcohol users. This comparison also suggests that the changes reported in this study are result of an active alcohol use rather than the status of HIV infection.

### Unchanged plasma cytokine/chemokine levels in HIV-infected alcohol users

**Fig. 4:**
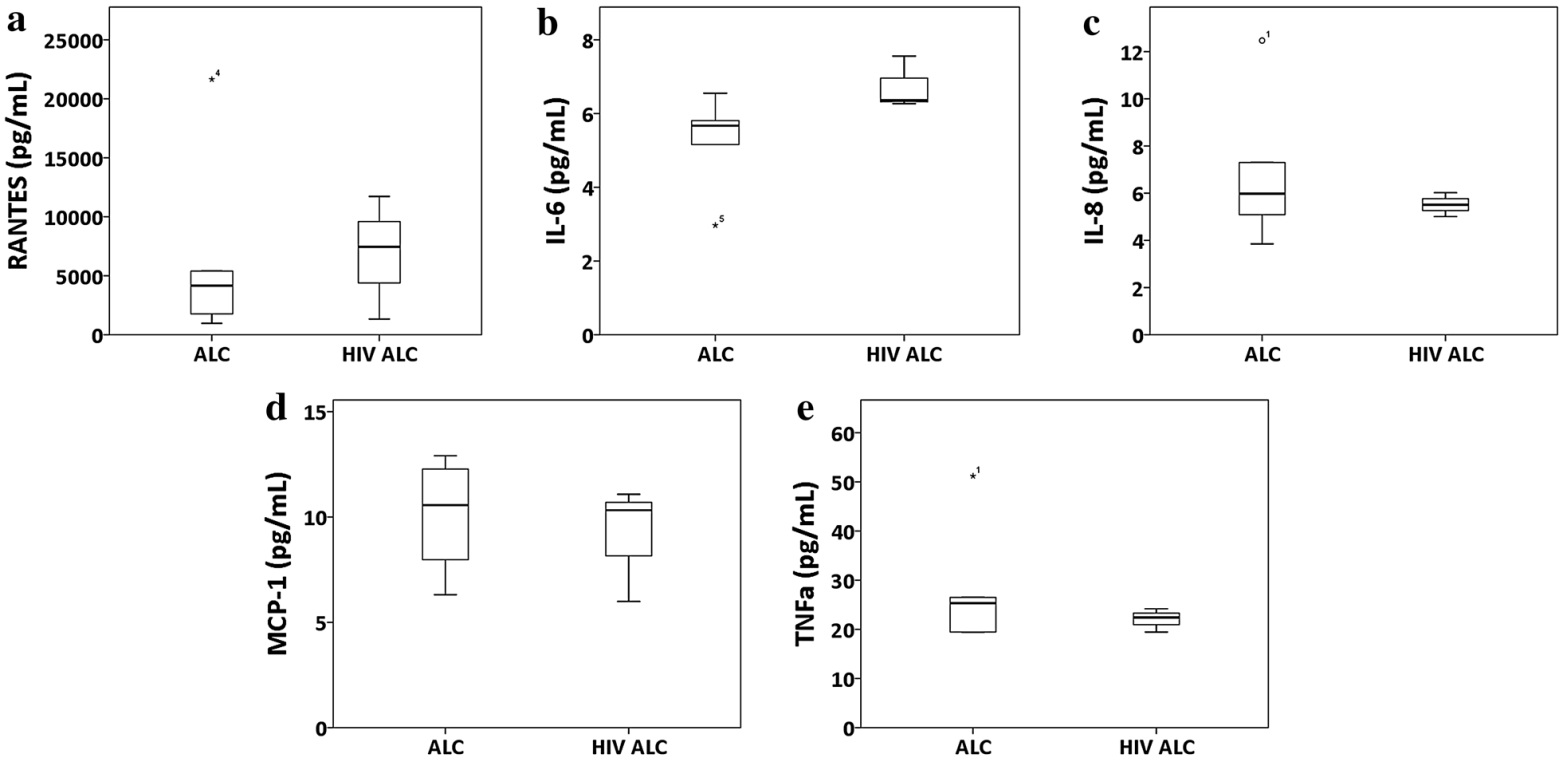
Plasma concentration of cytokines/chemokines in HIV negative alcohol users (ALC; n = 6) and HIV positive alcohol users (HIV+ ALC; n = 4). **a** RANTES, **b** IL-6, **c** IL-8, **d** MCP-1, and **e** TNF-α between. The analysis was done using *box* and *whisker plots*. The *box* represents the 25th–75th quartile, the *whiskers* represent the range of values, the median is presented as a *line* inside the *box*, and the out of range values are presented as *circles* or *stars* *above* and *below* the *whiskers*

Cross-talk between proinflammatory cytokines/chemokines, oxidative stress, and CYP have been suggested in previous studies [31, 32]. Hence, to evaluate the contribution of proinflammatory cytokine towards the observed enhanced oxidative stress in the HIV positive user group, as compared to the HIV negative alcohol user group, the plasma levels of important inflammatory cytokines/chemokines were determined in this study. As shown in Fig. 4, the plasma levels of the analyzed cytokines/chemokines—regulated on activation, normal T cell expressed and secreted (RANTES), interleukin (IL)-6, IL-8, monocyte chemotactic protein-1 (MCP-1), and tumor necrosis factor-alpha (TNF-α)—remained unchanged between the HIV-negative and HIV positive user groups (p > 0.05). Similar trends with the levels of these cytokines/chemokines were observed in HIV-infected non-alcohol users [19].

Cytokines have been shown to play a role in HIV pathogenesis [33]. Previous studies have also demonstrated alcohol consumption to be associated with an increased production of proinflammatory chemokine/cytokines [34, 35]. The results from this study suggests that the status of HIV infection has no additional effect on the existing plasma levels of RANTES, IL-6, IL-8, MCP-1, and TNF-α. This suggests the observed enhanced oxidative stress in HIV positive alcohol users may be mediated by pathways independent of the cytokines/chemokines analyzed in this study.

## Conclusions

In conclusion, this is the first clinical report on the increase in oxidative stress in HIV positive alcohol users. Since alcohol and oxidative stress independently have been implicated in increased HIV replication, it is possible that alcohol exacerbates HIV pathogenesis through an oxidative stress pathway. In addition, our study suggests possible involvement of CYP2E1 enzyme and a pathway that regulates AOEs in modulating oxidative stress induced by alcohol in HIV positive patients. We speculate that increased CYP2E1 expression upon chronic use of alcohol increases alcohol-induced oxidative stress. Furthermore, increased chronic use of alcohol may also reduce the antioxidant properties of monocytes leading to an exacerbation in oxidative stress. Further evaluation of CYP and AOEs pathways in alcohol-mediated HIV pathogenesis may provide novel therapeutic targets to treat HIV-infected alcohol users more effectively.

## Methods

### Study subjects

The recruitment and consent procedure for this study was approved by both the Institutional Review Board (IRB) from the University of Missouri-Kansas City, Kansas City, MO and the IRB/Institutional Ethic Committee (IEC) from Provincial Regional Hospital, Ministry of Public Health, Bamenda, Cameroon.

The subjects for this study were recruited in Cameroon, Africa in order to acquire sufficient numbers of subjects who are not already receiving ART at the time of sample collection. HIV-infected patients in the USA usually initiate ART as soon as they are confirmed positive for HIV, and therefore, it is very difficult to recruit ART-naïve patients in USA. After exclusions, ten ART-naïve human subjects were recruited and assigned to two different cohorts—(a) healthy HIV negative control subjects who reported mild-to-moderate alcohol use (ALC; n = 6) and (b) HIV positive mild-to-moderate alcohol user (HIV+ ALC; n = 4). Based on power analysis, 6–8 human subjects were required to obtain a power of ≥0.8 [19]. However, our recent reports [19, 20] from the same number of subjects for HIV+ Smokers (n = 4) suggested that 4 subjects were sufficient to obtain significant (p ≤ 0.05) and borderline significant (p ≤ 0.1) differences between these groups. In addition, it was extremely difficult to obtain HIV-infected subjects who were also alcohol users in Cameroon, Africa because patients are strongly advised to stop drinking alcohol immediately they are determined to be HIV positive. Informed written consent was obtained from all participants.

#### Inclusion and exclusion criteria

The inclusion and exclusion criteria for the recruitment were similar to our recent study [19]. In brief, participants between the ages of 31–51 years were recruited for this study, because relatively younger and older subjects have decreased metabolic activity and antioxidant defense. In addition, this age group has high prevalence in terms of alcohol use and HIV-infection. Alcohol consumption criteria for inclusion were 7–14 drinks/week for men and 4–7 drinks/week for women as defined by the National Institute on Alcohol Abuse and Alcoholism (NIAAA). The recruited subjects were mild-to-moderate alcohol users for the past 6 months. With respect to the HIV positive category, individuals with CD4+ counts between 200 and 500 cells/µl were enrolled. The CD+ counts for the HIV-negative category (ALC) was >500. Participants who were recruited and excluded using the following strict criteria were: (1) pregnant/lactating women; (2) subjects with infectious diseases like tuberculosis and hepatitis A/B/C; (3) subjects receiving ART or on other CYP2E1-affecting medications to minimize the effects of drugs on alcohol metabolism; (4) subjects who consume other recreational substances of abuse, e.g. methamphetamine, cocaine, or marijuana; (5) moderate-to-heavy cigarette smokers (>1 pack per day). Upon applying these strict criteria, recruitment of subjects was challenging and the final selection was based on a personal interview, analysis of health history, and screening for other infectious diseases such as malaria.

### Collection of peripheral blood mononuclear cells and monocytes

Peripheral blood mononuclear cells (PBMC) and monocytes were collected as described previously [19]. Briefly, Ficoll Hypaque plus was used to collect and isolate PBMC, and Dynabeads FlowComp human CD14 kit (Invitrogen, Grand Island, New York) was used to fractionate CD14+ monocytes.

### Quantitation of oxidative DNA damage and glutathione levels

The plasma concentration of 8-hydroxy-2′-deoxyguanosine (8-OHdG) was determined using oxiselect oxidative DNA damage ELISA kit (Cell Biolabs, San Diego, CA, USA) as described previously [19]. Briefly, following dilution of the plasma (1:1), the samples were added to an 8-OHdG/BSA conjugate on a preabsorbed EIA plate followed by anti-8-OHdG primary and HRP conjugated secondary antibodies. The reaction was terminated after 1 h incubation and the absorbance was measured at 450 nm using a spectrophotometer. The 8-OHdG content in monocytes was determined using the EpiQuik 8-OHdG DNA Damage Quantification Direct Kit (Fluorometric) (Epigentek, Farmingdale, NY, USA). As per the manufacturer’s protocol 100–300 ng of sample DNA was used, and 8-OHdG was detected using a fluorescence microplate reader at 530_ex_/590_em_ nm. The amount of 8-OHdG was calculated using a standard curve generated from the standard solution provided in the kit. The levels of oxidized and reduced glutathione in plasma was measured using the kit from BioVision Inc (Milpitas, CA, USA) following manufacturer’s protocol.

### Plasma alcohol levels

The plasma alcohol concentration was determined using the ethanol assay kit from BioVision Inc. (Milpitas, CA, USA) following manufacturer’s instruction. Briefly, the plasma samples were diluted with assay buffer (10–100 fold) and incubated with alcohol oxidase and detector probe for 1 h. The absorbance was measured at 570 nm and test samples were compared with the standard to determine the alcohol concentration.

### DNA/RNA/Protein collection

DNA, RNA, and protein were isolated from monocytes using the All prep DNA/RNA/Protein QIAGEN Kit (Valencia, CA, USA). Briefly, the samples were lysed in RLT buffer, transferred to the DNA column, and centrifuged to collect the flow through. The DNA was first collected using DNA elution buffer, followed by RNA using RNA elution buffer. Protein was collected upon precipitating the RNA sample using APP buffer. The RNA and DNA were quantified using NanoDrop (Thermo scientific, Rockford, IL, USA). The protein was quantified using the Pierce BCA protein assay kit (Life technologies, Grand Island, NY, USA). However, the quantity of protein was very low and insufficient for analysis.

### RTPCR

qRTPCR was performed as described previously [19, 26]. Briefly, 60 ng of RNA was reverse transcribed to cDNA and then amplified using a two-step TaqMan Gene Expression Kit (Life technologies, Grand Island, NY, USA) in an iCycler iQ system (Bio-Rad Laboratories, Hercules, CA, USA) The gene expression levels were measured for the following CYP and AOEs using the probes obtained from Applied biosystems with the following ID numbers for these genes: CYP2E1 (Hs00559367_m1), Catalase (Hs00156308_m1), SOD1 (Hs00533490_m1), SOD2 (Hs00167309_m1), GSTK1 (Hs00210861_m1), and nuclear factor (erythroid-derived 2)-like 2 (NRF2) (Hs00232352_m1). As described previously, the relative fold expression for the gene of interest was calculated using the $2^{{ - \Delta \Delta {\text{C}}_{\text{t}} }}$ method by employing glyceraldehyde 3-phosphate dehydrogenase (GAPDH) as the housekeeping gene [19].

### Plasma cytokine levels

Plasma levels of various pro-inflammatory cytokines/chemokines including RANTES, IL-6, IL-8, MCP-1, and TNF-α were measured as described previously [19] using the multiplex cytokine assay kit (Bio-Rad, CA, USA). Briefly, plasma samples were centrifuged and the supernatants were diluted with sample diluent (1:3). Next, 50 µl of each sample was used to determine the levels of these cytokines/chemokines using Biorad Bioplex HTS (Bio-Rad, CA, USA). The levels of cytokines were determined using the standard curve.

### Statistical analysis

Statistical analyses were performed using the IBM SPSS software version 21. Comparisons among the two groups were conducted using independent student t test analysis. All statistical tests were two-sided and results obtained were considered borderline significant at p ≤ 0.1 (^#^) and significant at p ≤ 0.05 (*).

## Abbreviations

ART: antiretroviral therapy
CYP: cytochrome P450
AOEs: antioxidant enzymes
ROS: reactive oxygen species
SOD: superoxide dismutase
GSTK1: glutathione S-transferase kappa 1
GSH: glutathione
GSSG: glutathione disulfide
NRF2: nuclear factor (erythroid-derived 2)-like 2
8-OHdG: 8-hydroxy-2′-deoxyguanosine
RANTES: regulated on activation normal T cell expressed and secreted
IL: interleukin
MCP-1: monocyte chemotactic protein-1
TNF-α: tumor necrosis factor-alpha
